# Genetic association of Interleukin-17A polymorphism in Bangladeshi patients with breast and cervical cancer: a case-control study with functional analysis

**DOI:** 10.1186/s12885-024-12352-0

**Published:** 2024-05-30

**Authors:** Md. Abdul Aziz, Subrina Chowdhury, Sarah Jafrin, Md Abdul Barek, Mohammad Sarowar Uddin, Md. Shalahuddin Millat, Mohammad Safiqul Islam

**Affiliations:** 1 Department of Pharmacy, Faculty of Science, Noakhali Science and Technology University, Noakhali, 3814 Bangladesh; 2 Laboratory of Pharmacogenomics and Molecular Biology, Department of Pharmacy, Noakhali Science and Technology University, Noakhali, 3814 Bangladesh; 3 Bangladesh Pharmacogenomics Research Network (BdPGRN), Dhaka, 1219 Bangladesh

**Keywords:** Interleukin-17A, *IL-17A*, Breast cancer, Cervical cancer, Association, Correlation, Case-control, Polymorphism

## Abstract

**Background:**

Breast and cervical cancer are the two leading cancers in terms of incidence and mortality. Previous studies reported different interleukins, including interleukin-17A (*IL-17A*) to be responsible for the development and progression of these malignancies. Therefore, we speculated that the variants in this gene might be associated with these cancer developments in Bangladeshi population. For evaluating the hypothesis, we investigated the association of *IL-17A* rs3748067 polymorphism with the susceptibility of both breast and cervical cancer.

**Methods:**

This case-control study was performed on 156 breast cancer patients, 156 cervical cancer patients, and 156 controls using the tetra-primer amplification refractory mutation system-polymerase chain reaction. The statistical software package SPSS (version 25.0) was applied for analyses. The genetic association was measured by the odds ratio (OR) and 95% confidence intervals (CIs). A statistically significant association was considered when *p*-value ≤ 0.05. Functional analysis was performed using GEPIA and UALCAN databases.

**Results:**

From the calculation of the association of *IL-17A* rs3748067 with breast cancer, it is found that no genotype or allele showed a statistically significant association (*p*>0.05). On the other hand, the analysis of *IL-17A* rs3748067 with cervical cancer demonstrated that CT genotype showed a significant association (CT vs. CC: OR=1.79, *p*=0.021). In the overdominant model, CT genotype also revealed a statistically significant association with cervical cancer, which is found to be statistically significant (OR=1.84, *p*=0.015).

**Conclusion:**

Our study summarizes that rs3748067 polymorphism in the *IL-17A* gene may be associated with cervical cancer but not breast cancer in Bangladeshi patients. However, we suggest studies in the future with a larger sample size.

**Supplementary Information:**

The online version contains supplementary material available at 10.1186/s12885-024-12352-0.

## Background

 Breast cancer became the leading cancer worldwide in 2020, with a reported 2.3 million new cases representing 11.7% of total cancer incidence. In terms of mortality, breast cancer is the fifth leading causes of mortality globally, with approximately 685,000 deaths [1–3]. Cervical cancer, in contrast, is the fourth most diagnosed malignancy and also the fourth major cause of mortality in females. In 2020 alone, about 604,000 new cervical cancer cases and 342,000 deaths were reported. Moreover, cervical cancer was found to be one of the top three cancers that affect females under the age of 45 in 146 countries, which accounts for 79% in 185 countries assessed [2, 4].

Patients’ age, reproductive and hormonal factors (first birth or menarche at early age, fewer children, less breastfeeding, menopause at later age, menopausal hormone therapy, and oral contraceptives), personal or family history, genetic predisposition, environmental factors, and lifestyle factors (alcohol consumption, excessive body weight, and physical inactivity) have been correlated with an elevated risk for the development and progression of breast cancer [5–7]. Again, risk factors of cervical malignancy include both behavioral (sexual activity and lifestyle factors) and certain infectious (human papillomavirus) contributors [8]. Other risk factors are age at the first full-term pregnancy, diet, family history, immunosuppression, immune deficiency, oral contraceptives, parity, and smoking [9–11].

Interleukin-17 A (IL-17A) is one of the most intensively investigated interleukins from the IL-17 family which play a critical function in cancer development, progression, and control [12, 13]. It is found in the human chromosome 6.12.2 and encodes a 155 amino acid containing protein (consisting of signal peptide with 23 amino acids and a mature peptide with 132 amino acids) [14]. In carcinogenesis, IL-17A has been reported to engage myeloid-derived suppressor cells (MDSCs) that repress anti-tumor activity [15, 16]. IL-17A could also stimulate unnecessary tumor growth by influencing IL-6, which in turn activates tumorigenic signal transducer and activator of transcription (STAT3) signaling pathway and over-express genes associated with pro-survival and pro-angiogenesis [17].

Numerous studies have reported a higher expression of IL-17A in tumor cells, including breast cancer, colorectal carcinoma, gastric carcinoma, hepatocellular carcinoma, ovarian cancer, medulloblastoma, pancreatic cancer, non-small-cell lung cancer, and thyroid cancer [18, 19]. Polymorphisms in the *IL-17A* gene have been investigated over time to find the possible association with cancers. A major single nucleotide polymorphism (SNP) in the *IL-17A* gene is rs3748067 which is found on the 3’-untranslated regions (UTR) in chromosome 6 location 52,190,541. The association of r3748067 polymorphism with various cancers has been extensively evaluated in the last decade that includes breast cancer [20], cervical cancer [21–26], colorectal cancer [27, 28], gastric cancer [29, 30], lung cancer [31], and others.

Although previous studies have evaluated the correlation of *IL-17A* gene rs3748067 polymorphism with the susceptibility of breast and cervical cancers, the results were incosistent. Besides, no study has been performed in Bangladeshi breast and cervical cancer patients to evaluate the association of rs3748067 polymorphism. Therefore, we conducted the present case-control study to analyze the association of the common SNP in the *IL-17 A* (rs3748067) gene with the susceptibleness of breast and cervical cancer.

## Methods

### Study settings

The reporting of the present retrospective case-control analysis conforms to the latest STROBE guidelines designed for case-control studies [32]. In this study, we recruited two groups of patients: one group with breast cancer and another group with cervical cancer. Both groups consisted of 156 patients, each of whom was appointed randomly from the National Institute of Cancer Research and Hospital (NICRH) during the period from July 2019 to June 2020. Again, for the control arm, we recruited 156 healthy volunteers, who visited the NICRH during the time of patient recruitment by matching their age and sex with the breast and cervical cancer patients. A predesigned study protocol and a consent form were used for the clinical investigation of breast and cervical cancer patients. Ethical permissions were obtained from the NIRCH (for breast cancer: NICRH/Ethics/2019/446 and for cervical cancer: NICRH/Ethics/2019/447) ethics committee. We used a standard questionnaire for collecting the details of the patients, including their sociodemographic details, clinicopathological history, and present status. Sociodemographic details of the controls were also recorded. We have selected patients who were free from other comorbidities such as liver, lung, and kidney diseases. This study was conducted at the Laboratory of Pharmacogenomics and Molecular Biology located at the Department of Pharmacy, Noakhali Science and Technology University.

### Blood sample collection and DNA extraction

Each participant included in this case-control study donated about 3 ml of blood. The blood samples were collected via a 3 ml intact syringe and transferred immediately into an ethylene diamine tetra acetic acid (EDTA) containing plastic tube. The tubes were then stored in a -80ºC refrigerator until processed. The extraction of genomic DNA from whole blood was completed following the DNA extraction method described by Islam and colleagues [33] using a DNA extraction kit provided by Favorgen (Taiwan). The purity of extracted DNA was assessed by keeping the absorbance ratio A260:A280 and samples with a ratio of > 1.5 were considered pure DNA.

### Primer design and genotyping

**Fig. 1:**
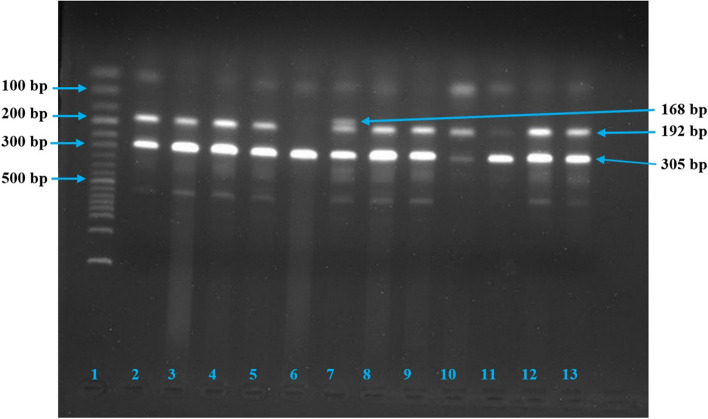
PCR products for *IL-17A* rs3748067 in breast cancer after 1.5% agarose gel electrophoresis

**Fig. 2:**
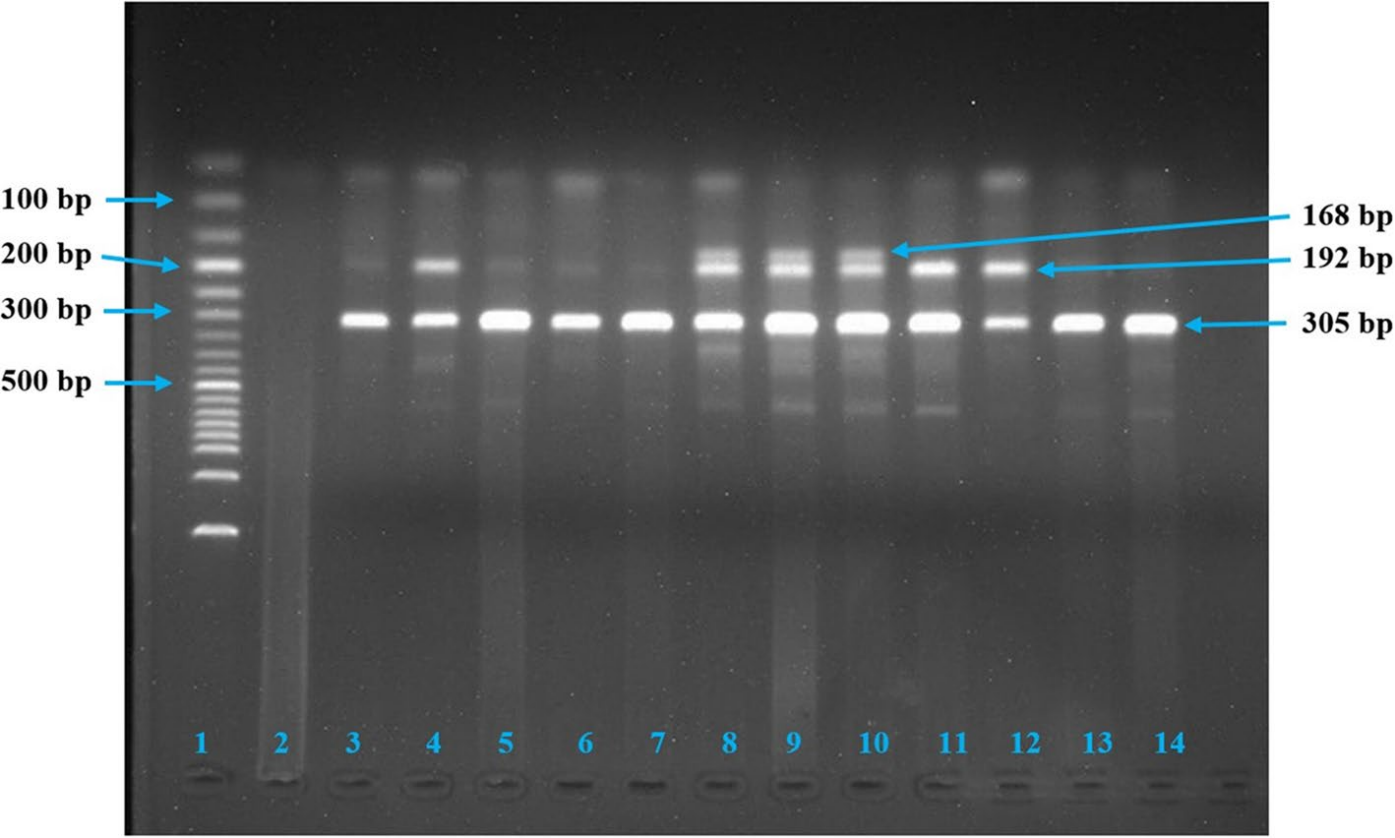
PCR products for *IL-17A* rs3748067 in cervical cancer after 1.5% agarose gel electrophoresis

There are different online-based software available for designing primers. We have used the Primer1 software to design four required primers. For genotyping process, the tetra-primer amplification refractory mutation system–polymerase chain reaction (T-ARMS–PCR) was utilized as described by Aziz and colleagues [34]. To validate the method, we first carried out a gradient PCR at temperatures ranging from 60°C to 65°C by a continuous alteration of primer concentration and MgCl_2_ concentration. After completing multiple PCRs, the intended PCR products for *IL-17A* rs3748067 were found at the temperature of 65°C. The genotyping of all samples was completed using the same formula of the PCR working master mix at the temperature of 65°C and visualized using ethidium bromide-stained 1.5% gel electrophoresis. The details of primers and conditions are listed in Table 1, and the agarose gel images are shown in Fig. 1 (breast cancer samples) and Fig. 2 (cervical cancer samples). For controlling the quality of genotyping and ensuring repeatability, 20% of the samples were randomly assessed.

**Table 1:** Primer sequences and PCR conditions with observed products

Gene	SNP	Primers (5’-3’)	% GC	PCR conditions	No. of cycles	Size of PCR products (bp)
*IL-17A*	rs3748067	**FI**: CTTGGGCTGAACTT TTCTCATACTTACAG **RI**: AAAGGAGCTGAT GGGGCAGAACGCAT **FO**: TCTAGAGGCCTTC AGAAGTAGGGCAAGA **RO**: GTCCAGTTTCTCC CCTAGACTCAGGCTT	41.4 53.8 50.0 53.6	95°C for 5 min 95°C for 1 min 65°C for 45 s 72°C for 1 min 72°C for 10 min	35	CC: 192, 305 CT: 168, 192, 305 TT: 168, 305

*SNP *Single nucleotide polymorphism, *PCR *Polymerase chain reaction, *FI *Forward inner, *RI *Reverse inner, *FO *Forward outer, *RO *Reverse outer, *BP *Base-pair

### Statistical analysis

The sociodemographic and clinicopathological characteristics were reported as percentages. The genotypes and allele frequencies were measured for the deviation from the Hardy-Weinberg equilibrium (HWE) by applying the χ^2^-statistic. The link of *IL-17A* gene rs3748067 polymorphism with breast and cervical cancer was calculated by logistic regression according to five genotypic models (additive model 1, additive model 2, dominant model, recessive model, and overdominant model) and allele model using odds ratio (OR) with 95% confidence intervals (CI). Best-fit model was determined using Akaike’s information criterion (AIC) and Bayesian information criterion (BIC) values. A statistically significant risk was considered in terms of *p*-value ≤ 0.05. Statistical calculations were done by the use of latest SPSS software version 25 (IBM Corp., Armonk, NY, USA).

### Functional analysis

The Gene Expression Profiling Interactive Analysis (GEPIA) (available at http://gepia.cancer-pku.cn) is a recently developed interactive web resource. It is utilized for examining the RNA sequencing expression data of 9,736 tumors and 8,587 normal tissues retrieved from The Cancer Genome Atlas (TCGA) along with the Genotype-Tissue Expression (GTEx) applying a standard processing pipeline. In this study, GEPIA was applied to evaluate the transcriptional level of the *IL-17A* gene expression of breast and cervical cancer tissues versus normal tissues and visualized using box plots. Hub genes with |Log_2_FC| ≥ 1 as well as *p* ≤ 0.05 were considered statistically significant. Again, the UALCAN is a comprehensive interactive web server for investigating cancer OMICS data [35]. We have used this webserver to show the *IL-17A* expression based on the sample types, patient’s age, individual cancer stages, patient’s race, weight, and tumor grade for both breast and cervical cancer.

## Results

### Characteristics of participants

The distribution of characteristic variables of breast cancer patients and healthy subjects is listed in Table 2. It is found that 40.38% of patients were under 45 years old and 46.15% were between 45 and 60 years. In comparison with cases, controls consisted of 50.64% under 45 years, and 43.58% were between 45 and 60 years. The average age of the breast cancer patients and controls was 45.37 years and 40.02 years, respectively. Besides, the average body mass index (BMI) was 28.61 kg/m^2^ and 22.57 kg/m^2^ in the patient group and control group, respectively. About 97.44% of cases were married, and 92.95% of controls were married. Of the patients, 61.21% had invasive duct cell carcinoma and most of the patients had grade II breast cancer consisting of 65.45%. Around 56.83%, 41.01%, 22.30%, 19.42%, and 17.27% of patients have been diagnosed by USG, biopsy, CT, FNAC, and X-ray, respectively. Almost half of the patients received surgery and around 71% of patients received 4–8 cycles of chemotherapy. Patients with negative hormonal status were prevalent such as 34.88% were ER (-), 34.11% were PR (-), and 39.53% were HER2 (-).

**Table 2:** Distribution of characteristic variables of breast cancer patients and controls

Variables		Breast Cancer Cases, *n *= 156 (%)	Controls *n* = 156 (%)
**Age (years)**	< 45	63 (40.38)	79 (50.64)
	45–60	72 (46.15)	68 (43.58)
	> 60	21 (13.46)	9 (5.77)
	45–60 + > 60	93 (59.61)	77 (49.36)
**Mean Age (years)**	Minimum Age	21	20
	Maximum Age	70	68
	Average	45.37	40.02
**BMI (kg/m** ^**2**^ **)**	Average	28.61	22.57
**Marital Status**	Married	152 (97.44)	145 (92.95)
	Unmarried	4 (2.56)	11 (7.05)
**Type of Breast Cancer**	Atypical ductal hyperplasia	1/116 (0.86)	N/A
	Duct cell carcinoma	5/116 (4.31)	
	Infiltrating duct cell carcinoma	30/116 (25.86)	
	Intraductal carcinoma	2/116 (1.72)	
	Invasive duct cell carcinoma	71/116 (61.21)	
	Medullary carcinoma	2/116 (1.72)	
	Metastatic duct cell carcinoma	4/116 (3.45)	
	Triple negative breast cancer	1/116 (0.86)	
	No data	40/156 (25.64)	
**Grade of Breast Cancer**	I	4/55 (7.27)	N/A
	II	36/55 (65.45)	
	III	15/55 (27.27)	
	No data	101/156 (64.74)	
**Diagnosis**	Biopsy	57/139 (41.01)	N/A
	CA 15.3	5/139 (3.60)	
	CT	31/139 (22.30)	
	CXR	2/139 (1.44)	
	Echocardiogram	6/139 (4.32)	
	FNAC	27/139 (19.42)	
	Lumpectomy	13/139 (9.35)	
	Mastectomy	13/139 (9.35)	
	RT	1/139 (0.72)	
	USG	79/139 (56.83)	
	X-Ray	24/139 (17.27)	
	No data	17/156 (10.90)	
**Current Treatment**	Chemotherapy	11/122 (9.02)	N/A
	CT	84/122 (68.85)	
	Mastectomy	4/122 (3.28)	
	MRM	2/122 (1.64)	
	RT	11/122 (9.02)	
	Surgery	10/122 (8.20)	
	No data	34/156 (21.79)	
**Previous Treatment**	CT	29/136 (21.32)	N/A
	Hormone therapy	10/136 (7.35)	
	Lumpectomy	4/136 (2.94)	
	Mastectomy	12/136 (8.82)	
	MRM	3/136 (2.21)	
	RT	39/136 (28.68)	
	Surgery	68/136 (50.00)	
	No data	20/156 (12.82)	
**Chemotherapy Cycle**	1–3	22/79 (27.85)	N/A
	4–8	56/79 (70.89)	
	9–12	1/79 (1.27)	
	No data	77/156 (49.36)	
**Hormonal Status**	ER (+)	38/129 (29.46)	N/A
	ER (-)	45/129 (34.88)	
	PR (+)	39/129 (30.23)	
	PR (-)	44/129 (34.11)	
	HER2 (+)	31/129 (24.03)	
	HER2 (-)	51/129 (39.53)	
	Triple negative	21/129 (16.28)	
	No data	27/156 (17.31)	

*BMI *Body mass index, *CT *Computed tomography, *MRM *Modified radical mastectomy, *USG *Ultrasound sonography, *RT *Radiotherapy, *FNAC *Fine-needle aspiration cytology, *CXR *Chest x-ray

The detailed characteristics of cervical carcinoma patients and healthy subjects are summarized in Table 3. As the data show, about 48.08% of patients were under 45 years old and 39.74% were between 45 and 60 years. The average age of cervical malignancy patients was 41.12 years, and the average BMI was 26.93 kg/m^2^. Approximately 94.87% of cases were married. The menstruation cycle starting age of 86.43% of patients was ≤ 13 years, whereas 90.28% of controls had their first menstruation cycle at ≤ 13 years. Again, the age of menstruation cycle stopping of 77.61% of patients was ≤ 45 years compared to 68.33% of the controls. Almost 80% of patients conceived their first child before or under 18 years, whereas 75.73% of controls gave birth to their first child at this age. The history of contraceptives shows that 75.64% of cervical cancer patients took pills and 63.56% of them took the pill for less than or equal to 5 years. Around 85% of cervical cancer patients had squamous cell carcinoma, and most of the patients were at IIB (56.55%) tumor stage, while 68.59% had grade 2 cancer and 55.13% were with T1 tumor size. 83.33% of patients were with negative (+) lymph nodes, and the status of distant metastasis showed that 68.42% of patients were in Mx state.

**Table 3:** Distribution of characteristic variables of cervical cancer patients and controls

Variables		Cervical Cancer Cases, *n* = 156 (%)	Controls *n *= 156 (%)
**Age (Years)**	< 45	75 (48.08)	79 (50.64)
	45–60	62 (39.74)	68 (43.58)
	> 60	19 (12.18)	9 (5.77)
	45–60 + > 60	81 (51.92)	77 (49.36)
**Mean Age (years)**	Minimum Age	20	20
	Maximum Age	73	68
	Average	41.12	40.02
**BMI (kg/m** ^**2**^ **)**	Average	26.93	22.57
**Marital Status**	Married	148 (94.87)	145 (92.95)
	Unmarried	8 (5.13)	11 (7.05)
**Menstruation Cycle Starting Age**	≤ 13	121/140 (86.43)	130/144 (90.28)
	> 13	19/140 (13.57)	14/144 (9.72)
	No data	16/156 (10.26)	12/156 (7.69)
**Menstruation Cycle Stopping Age**	≤ 45	52/67 (77.61)	41/60 (68.33)
	> 45	15/67 (22.39)	19/60 (31.67)
	No data	89/156 (57.05)	96/156 (61.54)
**First Child Conceived Age**	≤ 18	92/116 (79.31)	78/103 (75.73)
	> 18	24/116 (20.69)	25/103 (24.27)
	No data	40/156 (25.64)	53/156 (33.97)
**Age Gap Between 1st Child and 2nd Child**	≤ 2	96/102 (94.12)	82/90 (91.11)
	> 2	6/102 (5.88)	8/90 (8.89)
	No 2nd child	14/116 (12.07)	13/103 (12.62)
**Breastfeeding Period (Years)**	< 2	79/116 (68.10)	88/103 (85.44)
	≥ 2	37/116 (31.90)	15/103 (14.56)
**History of Taking Contraceptive Pills**	Yes	118/156 (75.64)	127/156 (81.41)
	No	38/156 (24.36)	29/156 (18.59)
**Taking Contraceptive Pills (Years)**	≤ 5	75/118 (63.56)	98/127 (77.16)
	> 5	43/118 (36.44)	29/127 (22.83)
**Postmenopausal Hormone Therapy**	Yes	0 (0.00)	N/A
	No	156 (100.00)	N/A
**Smoking History**	Yes	4/156 (2.56)	7/156 (4.49)
	No	152/156 (97.44)	149/156 (95.51)
**Type of Cancer**	Squamous cell carcinoma	132/156 (84.62)	N/A
	Adenocarcinoma	24/156 (15.38)	
**Tumor Stage**	I	13/145 (8.97)	N/A
	IIB	82/145 (56.55)	
	IIIA	7/145 (4.83)	
	IIIB	40/145 (27.59)	
	IVA	3/145 (2.07)	
	No data	11/156 (7.05)	
**Grade of Cancer**	Grade 1	40/156 (25.64)	N/A
	Grade 2	107/156 (68.59)	
	Grade 3	9/156 (5.77)	
**Tumor Size**	T1	86/156 (55.13)	N/A
	T2	52/156 (33.33)	
	T3	13/156 (8.33)	
	T4	5/156 (3.21)	
**Lymph Node Status**	Negative (-)	130/156 (83.33)	N/A
	Positive (+)	26/156 (16.67)	
**Nodal Status**	N1	60/96 (62.50)	N/A
	N2	29/96 (30.21)	
	N3	7/96 (7.29)	
	No data	60/156 (38.46)	
**Distant Metastasis**	Mx	104/152 (68.42)	N/A
	M0	37/152 (24.34)	
	M1	11/152 (7.24)	
	No data	4/156 (2.56)	

*BMI *Body mass index

### Distribution of genotypes of rs3748067

The frequency of genotypes in breast cancer patients obeyed HWE (χ^2^ = 2.85, *p*-value = 0.091) with a minor allele frequency of 20.51%. In controls, the genotype distribution did not show any deviation from HWE (χ^2^ = 3.46, *p*-value = 0.063) and minor allele frequency was 17.31%. The distribution of genotypes in cervical cancer patients also showed no departure from HWE (χ^2^ = 2.05, *p*-value = 0.152) and the frequency of minor allele was 21.15%, as shown in Table 4.

**Table 4:** Distribution of genotypes of *IL-17 A* rs3748067 in breast and cervical cancer cases and controls

Genotypes	Cases (*n* = 156) (%)	Hardy-Weinberg equilibrium (HWE)		Controls (*n* = 156) (%)	Hardy-Weinberg equilibrium (HWE)	
		χ^2^	*p*- value		χ^2^	*p*- value
Breast cancer						
CC	102 (65.38)	2.85	0.091	110 (70.51)	3.46	0.063
CT	44 (28.20)			38 (24.36)		
TT	10 (6.41)			8 (5.13)		
C	248 (79.49)			258 (82.69)		
T	64 (20.51)			54 (17.31)		
Cervical cancer						
CC	94 (60.23)	2.05	0.152	110 (70.51)	3.46	0.063
CT	58 (37.18)			38 (24.36)		
TT	4 (2.56)			8 (5.13)		
C	246 (78.85)			258 (82.69)		
T	66 (21.15)			54 (17.31)		

### Association between *IL-17A* rs3748067 variant with breast cancer

Table 5 presents the association analysis of *IL-17 A* gene rs3748067 polymorphism with breast cancer. From the analysis, it is found that additive model 1 and additive model 2 showed increased risk but the associations were not statistically significant (CT vs. CC: OR = 1.25, *p* = 0.394; TT vs. CC: OR = 1.35, *p* = 0.545, respectively). Other genotype models, such as dominant, recessive, and over-dominant models, also showed a similar nonsignificant association (CT + TT vs. CC: OR = 1.26, *p* = 0.332; OR = 1.27, *p* = 0.628; OR = 1.22, *p* = 0.441, respectively). In the allele model, minor allele T showed an enhanced risk association, and the association is not statistically significant (T vs. C: OR = 1.23, *p* = 0.307).

**Table 5:** Association of rs3748067 polymorphism with breast and cervical cancer

Genetic Models	Genotype/Allele	Cases, *N* = 156 (%)	Controls, *N* = 156 (%)	Crude Analysis			
				OR (95% Cl)	*p* -value	AIC	BIC
**Breast Cancer**							
Additive model 1 (CT vs. CC)	CC	102 (65.38)	110 (70.51)	1			
	CT	44 (28.20)	38 (24.36)	1.25 (0.75 to 2.08)	0.394	437.6	448.8
Additive model 2 (TT vs. CC)	CC	102 (65.38)	110 (70.51)	1			
	TT	10 (6.41)	8 (5.13)	1.35 (0.51 to 3.55)	0.545	437.6	448.8
Dominant model (CT + TT vs. CC)	CC	102 (65.38)	110 (70.51)	1			
	CT + TT	54 (34.62)	46 (29.49)	1.26 (0.79 to 2.04)	0.332	435.6	443.1
Recessive model (TT vs. CC + CT)	CC + CT	146 (93.59)	148 (94.87)	1			
	TT	10 (6.41)	8 (5.13)	1.27 (0.49 to 3.30)	0.628	436.3	443.8
Overdominant model (CT vs. CC + TT)	CC + TT	112 (71.79)	118 (75.64)	1			
	CT	44 (28.20)	38 (24.36)	1.22 (0.74 to 2.02)	0.441	435.9	443.4
Allele (T vs. C)	C	248 (79.49)	258 (82.69)	1			
	T	64 (20.51)	54 (17.31)	1.23 (0.82 to 1.84)	0.307		
**Cervical Cancer**							
Additive model 1 (CT vs. CC)	CC	94 (60.23)	110 (70.51)	1			
	CT	58 (37.18)	38 (24.36)	1.79 (1.09 to 2.92)	**0.021**	431.7	442.9
Additive model 2 (TT vs. CC)	CC	94 (60.23)	110 (70.51)	1			
	TT	4 (2.56)	8 (5.13)	0.58 (0.17 to 2.00)	0.394	431.7	442.9
Dominant model (CT + TT vs. CC)	CC	94 (60.23)	110 (70.51)	1			
	CT + TT	62 (39.74)	46 (29.49)	1.58 (0.98 to 2.52)	0.052	432.9	440.4
Recessive model (TT vs. CC + CT)	CC + CT	152 (97.44)	148 (94.87)	1			
	TT	4 (2.56)	8 (5.13)	0.49 (0.14 to 1.65)	0.248	435.1	442.6
Overdominant model (CT vs. CC + TT)	CC + TT	98 (62.82)	118 (75.64)	1			
	CT	58 (37.18)	38 (24.36)	1.84 (1.13 to 3.00)	**0.015**	430.5	438
Allele (T vs. C)	C	246 (78.85)	258 (82.69)	1			
	T	66 (21.15)	54 (17.31)	1.28 (0.86 to 1.91)	0.223		

*OR *Odds ratio, *CI *Confidence interval; *p*-value < 0.05 indicates statistically significant (bold), *AIC *Akaike information criterion, *BIC *Bayesian information criterion

### Association between *IL-17A* rs3748067 variant with cervical cancer

The correlation of *IL-17A* gene rs3748067 polymorphism with cervical cancer susceptibility (Table 5) demonstrated that two genetic association models, i.e., additive model 1 and over dominant model, showed a statistically significant association with cervical cancer (CT vs. CC: OR = 1.79, 95% CI = 1.09 to 2.92, *p* = 0.021; OR = 1.84, 95% CI = 1.13 to 3.00, *p* = 0.015). Other models did not show any significant association with cervical cancer (Additive model 2- TT vs. CC: OR = 0.58, *p* = 0.394; Dominant model - CT + TT vs. CC: OR = 1.58, *p* = 0.052; Recessive model: TT vs. CC + CT: OR = 0.49, *p* = 0.248; Allele model: T vs. C: OR = 1.28, *p* = 0.223).

### Comparison of genotypes and risk association between breast and cervical cancer

**Fig. 3:**
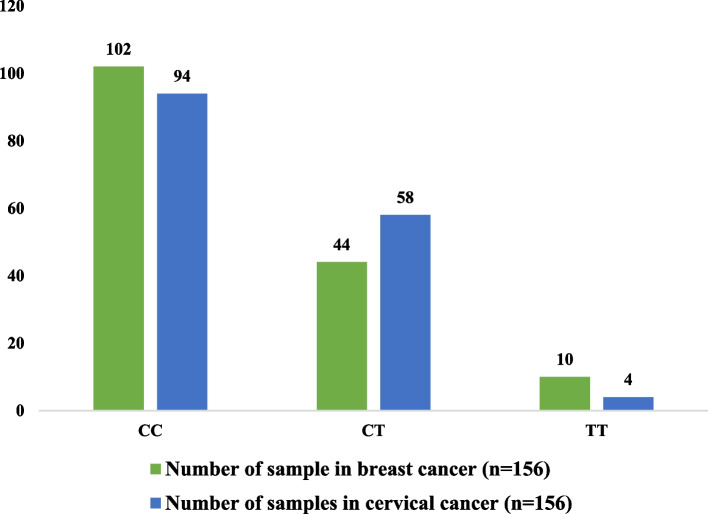
Comparison of genotypes of *IL-17 A* rs3748067 between breast and cervical cancer

The frequency of genotypes of *IL-17 A* rs3748067 and their comparison between breast cancer and cervical cancer patients are given in Fig. 3. It is observed that CC homozygote frequency (CC = 102) is higher in breast cancer patients than in cervical cancer patients (CC = 94). The distribution of CT heterozygote and TT mutant homozygotes shows that the frequencies of CT genotypes are higher, but TT genotypes are lower in cervical cancer patients (CT = 44 vs. 58 and TT = 10 vs. 4).

**Fig. 4:**
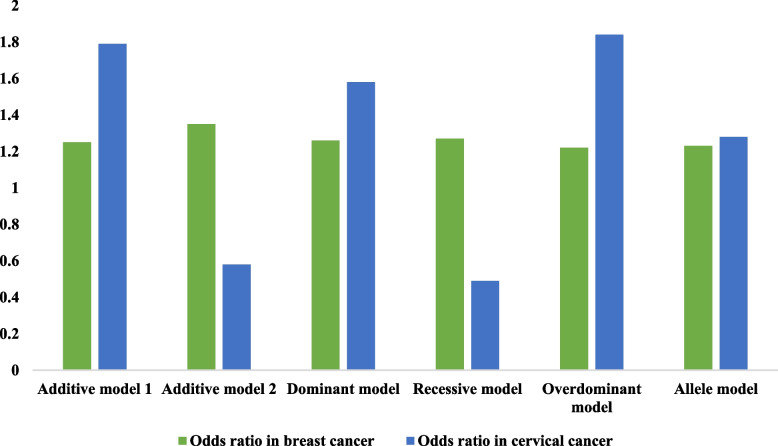
Comparison of risk association models of *IL-17A* rs3748067 between breast and cervical cancer population

Besides that, the comparison of ORs for analyzing the risk association of *IL-17A* rs3748067 between breast and cervical cancer patients (Fig. 4) showed that the ORs were higher for two genetic association models of cervical cancer- additive model 1 and overdominant model compared to breast cancer (1.79 vs. 1.25 and 1.84 vs. 1.22, respectively) and associations were also statistically significant. Although other genetic models in breast cancer showed higher ORs than in cervical cancer, except for the allele model, these models were not statistically significant. The model that produced the lowest values of AIC and BIC was deemed to be the optimal fit. It may be that the recessive model would be the most suitable choice for breast cancer, although no significant association was found, whereas, in the case of cervical cancer, the overdominant model is the best-fit model (Table 5).

#### IL-17A transcription levels

**Fig. 5:**
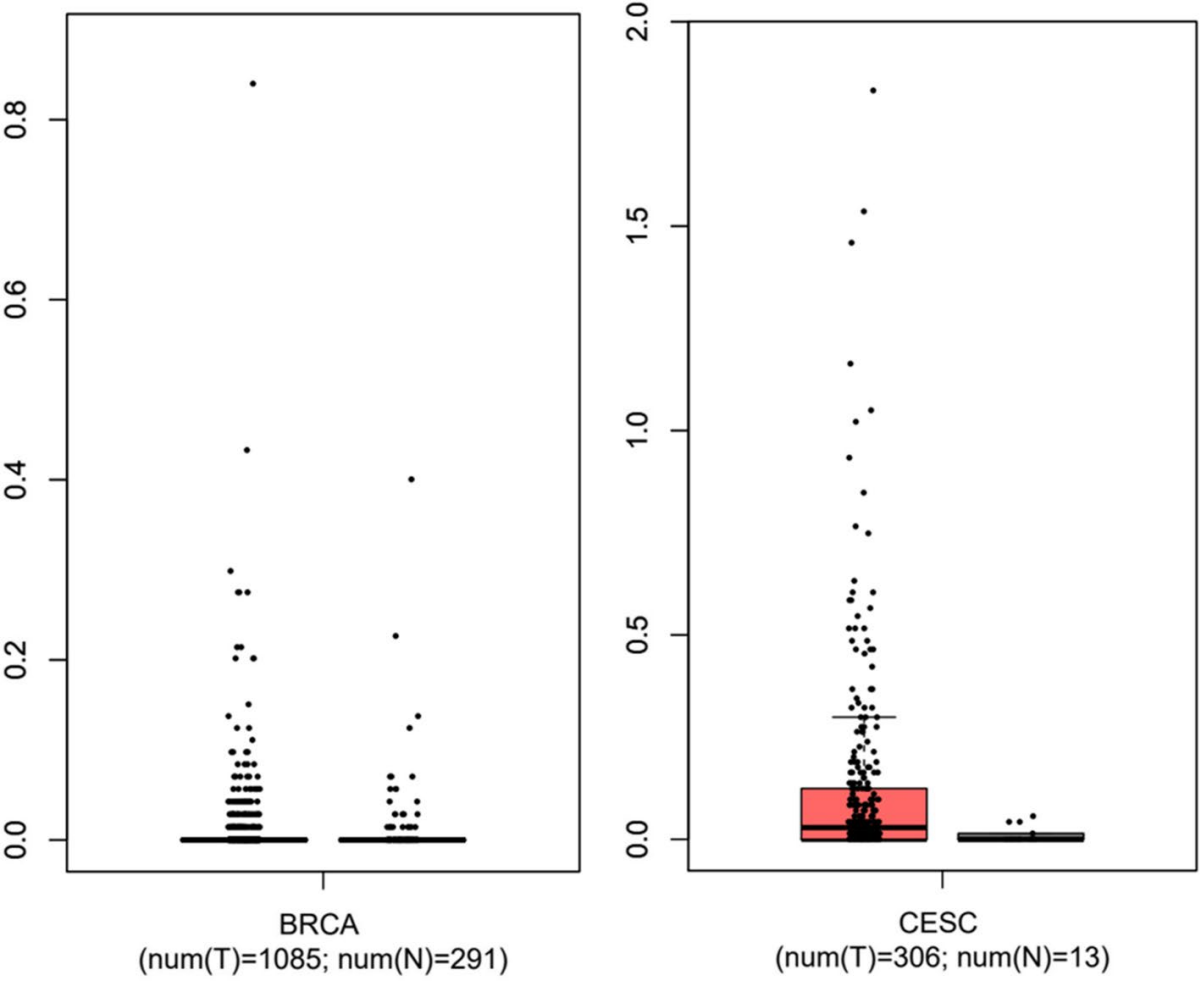
*IL-17A* gene expression of breast and cervical cancer tissues versus normal tissues based on the GEPIA (http://gepia.cancer-pku.cn)

The level of *IL-17A* transcription in breast and cervical cancer tissues versus normal tissues is visualized in Fig. 5. The box plots indicated that there is a significantly greater expression of *IL-17 A* in cervical carcinoma (CESC) tissues than in normal tissues. The expression level in breast carcinoma (BRCA) tissues and normal tissues was not statistically significant.

**Fig. 6:**
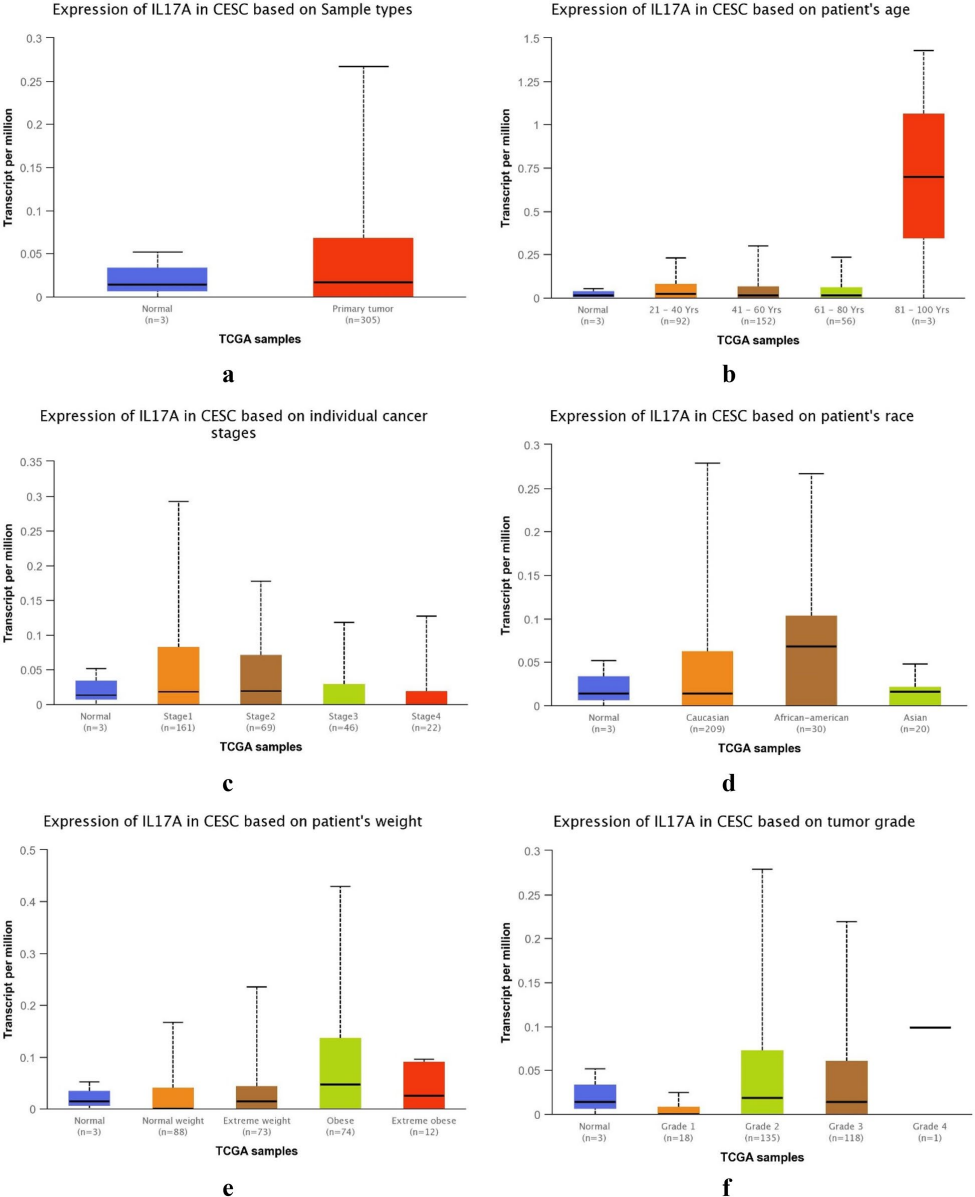
IL-17A expression based on the sample types, patient’s age, individual cancer stages, patient’s race, weight, and tumor grade of cervical cancer

The IL-17A expression based on sample types, patient’s age, individual cancer stages, patient’s race, weight, and tumor grade from the UALCAN web server for cervical cancer and breast cancer is depicted in Fig. 6 and Supplementary Fig. 1, respectively. The expression of IL-17A was found to be higher in cervical tumor samples (Fig. 6a), 81–100 years of age (Fig. 6b), cancer stage 1 and stage 2 (Fig. 6c), African American patients (Fig. 6d), obese and extremely obese patients (Fig. 6e), and tumor grade 2 and grade 3 patients (Fig. 6f). Again, in terms of breast cancer, no significant expression change was observed for sample types, patient’s age, cancer stages, patient’s race, and gender (Supplementary Fig. 7a-e) except for the medullary subtype (Supplementary Fig. 7f).

## Discussion

Breast and cervical cancers are the two most commonly diagnosed malignancies in females worldwide [1, 2]. Cytokines have been playing an indispensable role in tumor growth and progression. IL-17A is considered one of the most common cytokines from the IL-17 family that has been extensively studied due to its prominent role in carcinogenesis, especially in cervical and breast carcinoma besides inflammation [36]. A plethora of studies have described that IL-17A protein is greatly expressed within tumor tissues: for instance, gastric carcinoma, breast cancer, ovarian cancer, colorectal carcinoma, lung cancer, thyroid cancer, and hepatocellular carcinoma [18, 19]. Again, the increased IL-17A levels in the blood are linked with the aggressiveness of pancreatic adenocarcinoma, non-small cell lung cancer, thyroid tumors, laryngeal squamous cell carcinoma, and colorectal carcinoma [37–40]. In addition, previous studies with rs3748067 variant in the IL-17A gene described its notable association with a variety of cancers in multiple ethnicities, such as breast cancer [20], cervical cancer [21–26], colorectal cancer [27, 28, 41], gastric cancer [29, 30], and others. Based on the previous research, we performed this case-control study that reported the correlation of *IL-17A* rs3748067 polymorphism with the risk of cervical malignancy.

From the analysis in this study, we did not find any significant association of *IL-17 A* rs3748067 polymorphism with breast carcinoma in the studied population. However, our study found a higher frequency of the major allele C (79.49%) compared to the minor T allele (20.51). Again, the frequency of CC homozygous genotype was also greater (65.38%) than the heterozygous or mutant homozygous genotypes. The only previous study with this polymorphism in breast cancer also described similar findings. The study by Wang et al. (2012) showed no notable link between *IL-17A* gene rs3748067 variant and breast carcinoma in a Chinese case-control study in females. They also reported a higher frequency of a major allele (G allele) in their studied population [20]. To further explore the role of IL-17A in breast carcinoma, we analyzed the expression level in breast carcinoma (BRCA) tissues and normal tissues that were not statistically significant. Moreover, we did not observe any significant expression of IL-17A in terms of sample types, cancer stages, patient age, patient race, and gender.

Our study revealed a statistically significant link between *IL-17A* gene rs3748067 variant and cervical cancer. Our analysis demonstrated that CT genotype (OR = 1.79, *p* = 0.021) and over-dominant model (OR = 1.84, *p* = 0.015) are significantly correlated with cervical carcinoma risk. Besides, the frequency of the major allele (C allele: 78.85%) is greater than the minor allele (T allele: 21.15%). Moreover, we found that there is a significantly greater transcription level of *IL-17A* in cervical carcinoma (CESC) tissues than in normal tissues. The level of IL-17A expression was found to be higher in cervical tumor samples, 81–100 years of age, African American patients, obese and extremely obese patients, cancer stage 1 and stage 2, and in patients with tumor grade 2 and grade 3.

The correlation between *IL-17A* gene rs3748067 variant and breast carcinoma was examined for the first time in 2012 by Wang and colleagues [20] in Chinese Han women. The study recruited 491 breast cancer patients and 502 healthy individuals, and for genotyping, applied the SNaPshot technique. The study revealed that rs3748067 GG genotypes percentage was lower in PR-positive cases and was significantly correlated with PR hormonal status. They concluded that rs3748067 GG genotypes might be linked to poor prognosis and ineffective treatment. The link of this variant was not studied later in any other population. *IL-17A* rs3748067 has been studied in cervical cancer several times. The correlation was also evaluated in Chinese women by Niu et al. [22]. They explicated that subjects with the TT genotype and T allele were more prone to cervical carcinoma [22]. Another study in the Chinese population examined the contribution of rs3748067 in 352 cervical malignancy patients and 352 healthy controls using the PCR-RFLP method. However, they failed to establish any association between this polymorphism with cervical cancer [25]. Some other studies also failed to establish the association of rs3748067 variant with cervical carcinoma [24, 26].

The latest meta-analysis with rs3748067 variant in the *IL-17A* gene reported that it was correlated with cervical carcinoma, with T allele carriers depicting an enhanced risk [21]. Another meta-analysis conducted by Yang and colleagues [23] reported an elevated susceptibility of cervical carcinoma due to this polymorphism.

In this study, we have also tried to compare the frequency of genotypes of *IL-17A* rs3748067 between breast and cervical cancer patients. We have found that the frequency of CC homozygotes is greater in breast cancer patients than in cervical cancer patients. The distribution of CT heterozygote and TT mutant homozygotes reveals that the percentage of CT genotypes is higher, but TT genotypes are lower in cervical cancer patients. In addition, the comparison of ORs between breast and cervical cancer patients showed that the ORs were significantly higher for additive model 1 and the over-dominant model in cervical cancer compared to breast cancer.

It is to be mentioned that there are some limitations of the present study, such as the total number of participants included in the study is relatively low. Besides, all sociodemographic and clinicopathologic details of the participants were not possible to collect, which may alter the association. In addition, for this study, we have selected only available SNP from the public electronic database. However, our study has identified the link of *IL-17A* rs3748067 variant with cervical carcinoma and we are hopeful that our findings will have an impact on further studies that may result in stronger evidence. Besides these, possible interactions between the susceptibility loci and these risk factors should be thoroughly investigated.

## Conclusion

This study concludes that rs3748067 polymorphism in the *IL-17A* gene is associated with cervical cancer, not breast cancer in Bangladeshi patients. However, we suggest studies in the future with a larger sample size.

### Supplementary Information

**Supplementary Material 1.**

## Data Availability

The datasets used and/or analyzed during the current study are available from the corresponding author upon reasonable request. The details data cannot be shared publicly due to the restriction of the ethical committee.

## Abbreviations

IL-17A: Interleukin-17 A
SNP: Single nucleotide polymorphism
MDSCs: Myeloid-derived suppressor cells
STAT3: Signal transducer and activator of transcription
NICRH: National Institute of Cancer Research and Hospital
EDTA: Ethylene diamine tetra acetic acid
T-ARMS: Tetra-primer amplification refractory mutation system
PCR: Polymerase chain reaction
HWE: Hardy-Weinberg equilibrium
OR: Odds ratio
CI: Confidence intervals
GEPIA: Gene Expression Profiling Interactive Analysis
TCGA: The Cancer Genome Atlas
GTEx: Genotype-Tissue Expression
