# Supplementary material for: Genetic association of Interleukin-17A polymorphism in Bangladeshi patients with breast and cervical cancer: a case-control study with functional analysis
Source: BMC Cancer. 2024 May 30;24:660. doi: 10.1186/s12885-024-12352-0 (PMC11140929; doi:10.1186/s12885-024-12352-0)
Supplement: Supplementary file 1 — Supplementary Material 1. [file 12885_2024_12352_MOESM1_ESM.docx]

| 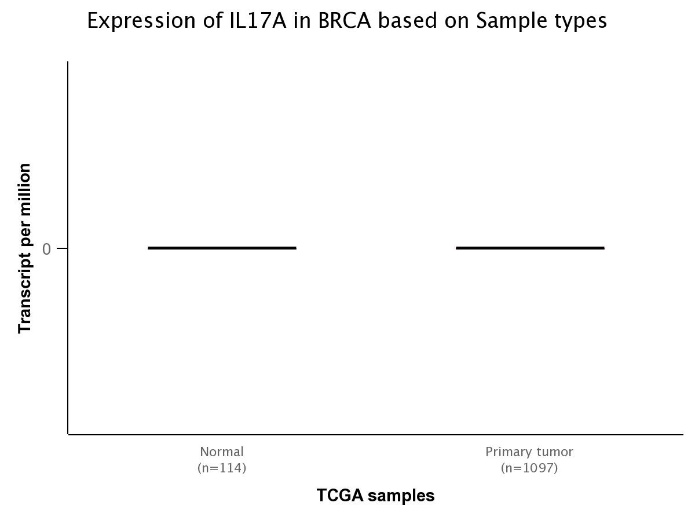 | 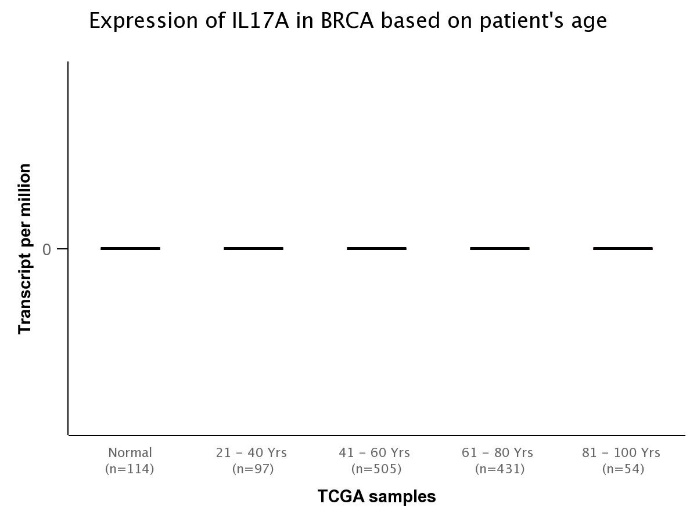 |
| --- | --- |
| **a** | **b** |
| 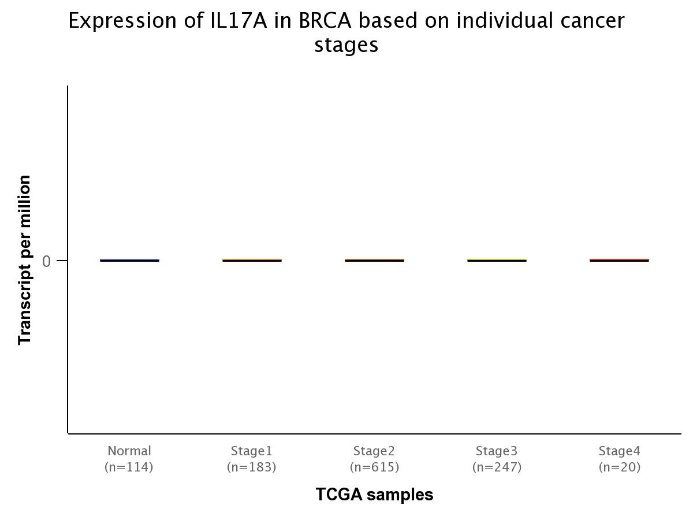 | 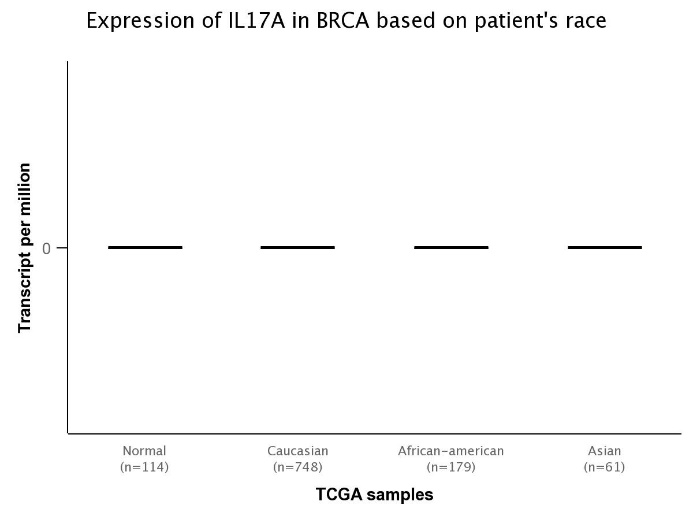 |
| **c** | **d** |
| 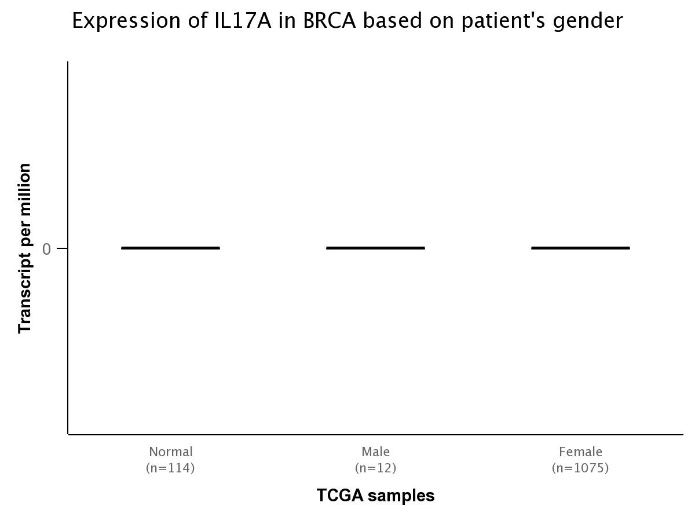 | 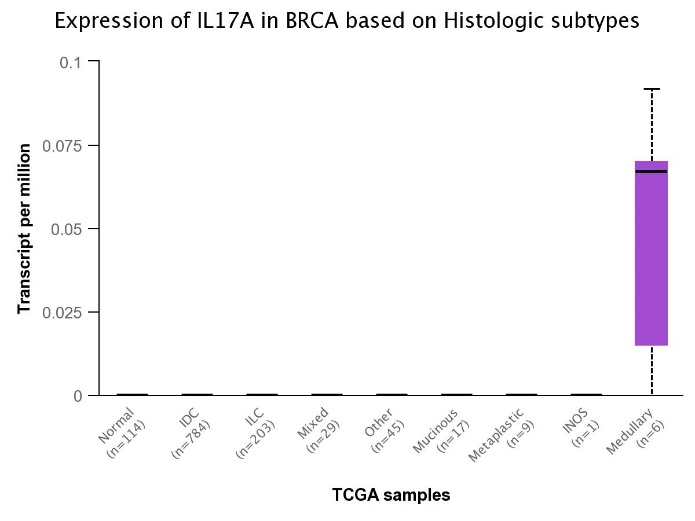 |
| **e** | **f** |

**Supplementary Fig. 1:** IL-17A expression based on the sample types, patient’s age, individual cancer stages, patient’s race, gender, and histologic subtypes of breast cancer.

**Raw Gel Figures**

**
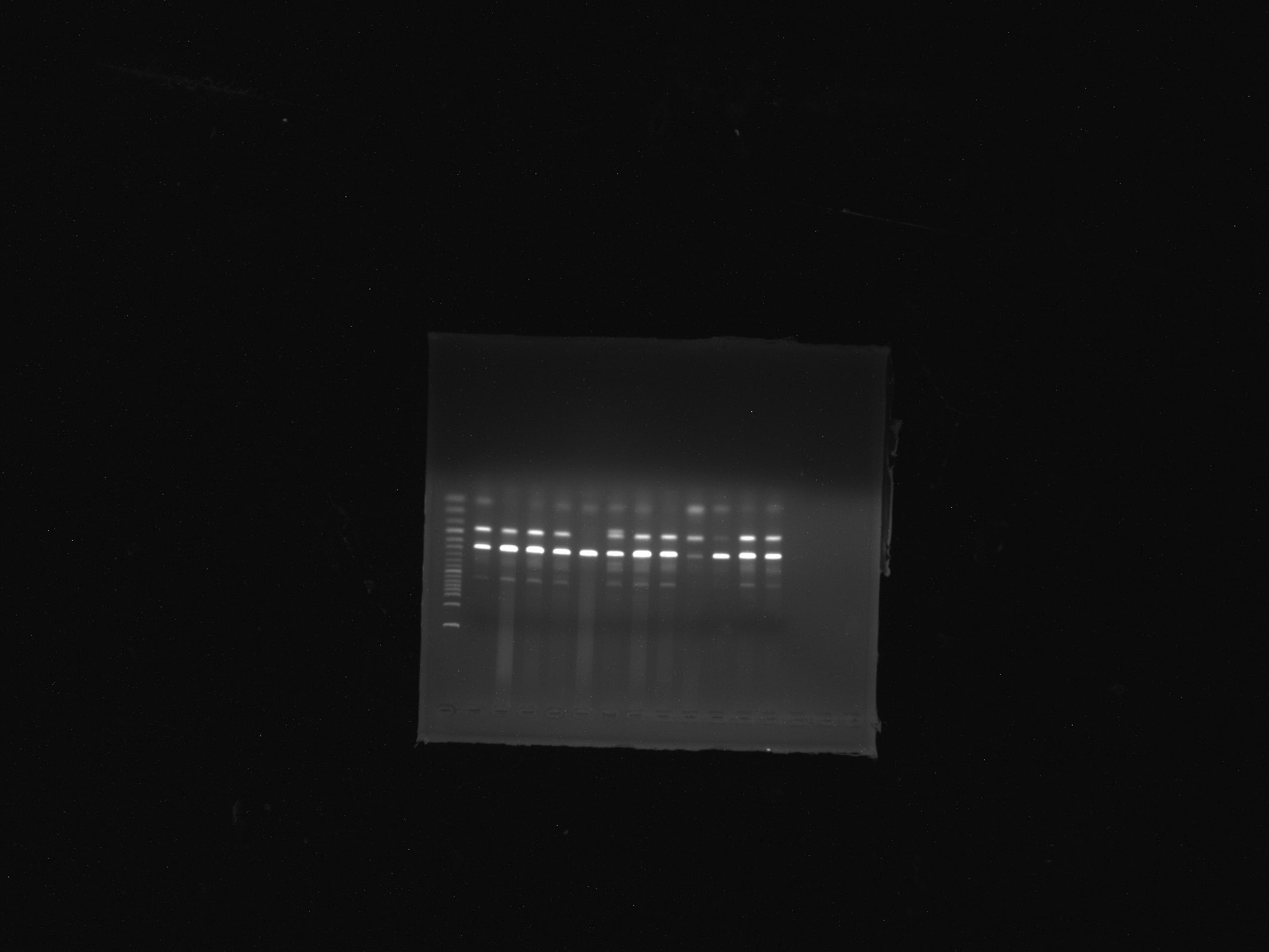
**

**Raw Gel Fig. 1**

**
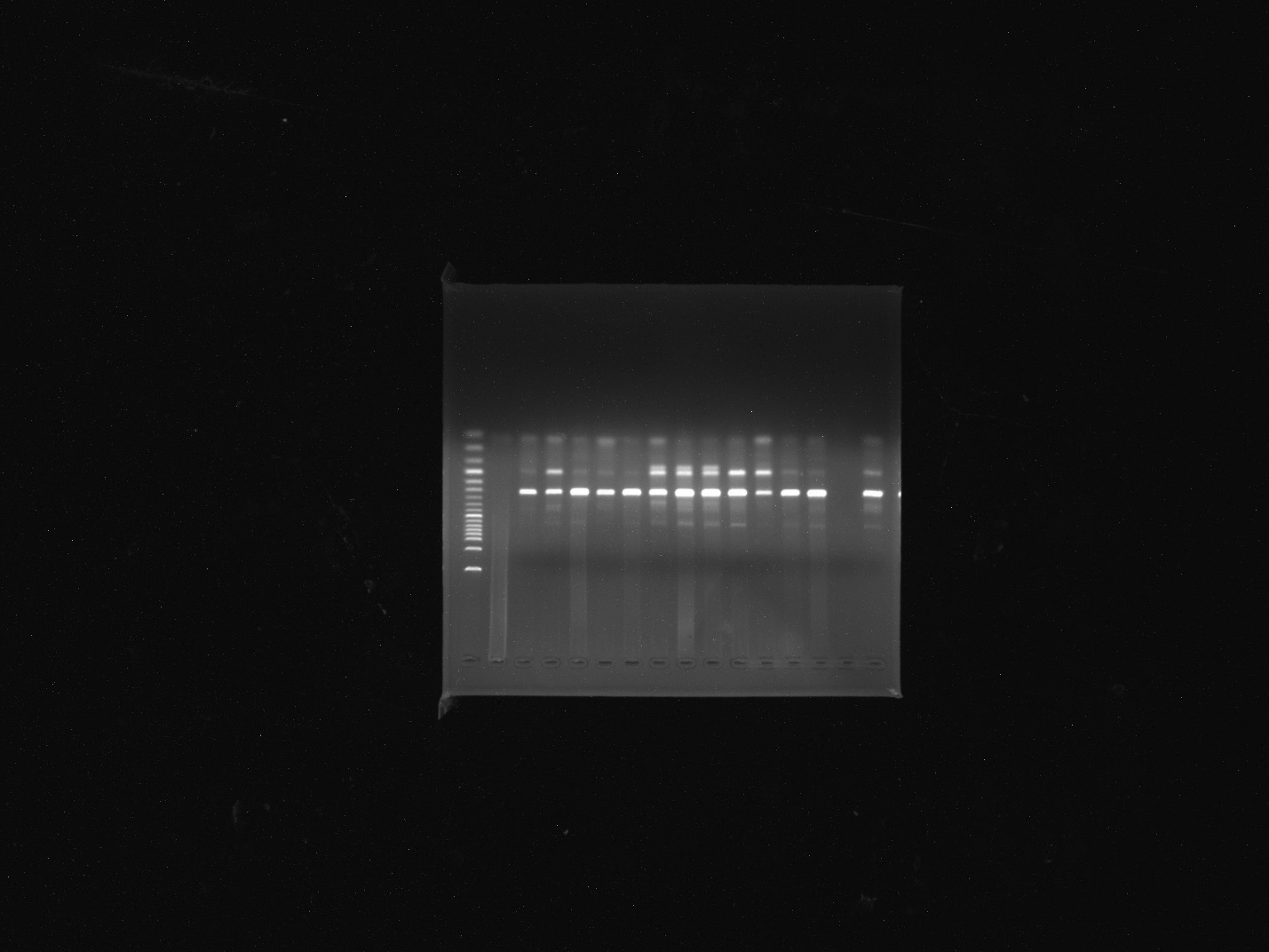
**

**Raw Gel Fig. 2**
